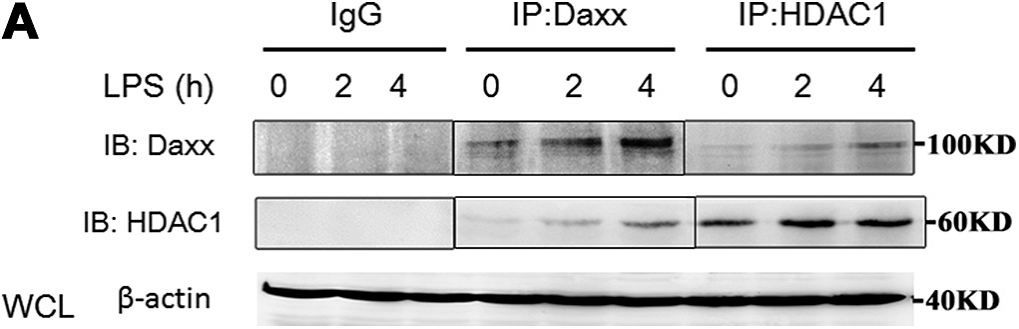# Correction: Death domain-associated protein 6 (Daxx) selectively represses IL-6 transcription through histone deacetylase 1 (HDAC1)-mediated histone deacetylation in macrophages

**DOI:** 10.1016/j.jbc.2021.101260

**Published:** 2021-10-05

**Authors:** Zhenyu Yao, Qian Zhang, Xia Li, Dezhi Zhao, Yiqi Liu, Kai Zhao, Yin Liu, Chunmei Wang, Minghong Jiang, Nan Li, Xuetao Cao

In Fig. 5*A*, the IL-12p40 panel was duplicated in the IFN-beta panel. Fig. 6*A* had splicing in the immunoblots between lanes three and four as well as lanes six and seven. The errors have been corrected: in Fig. 5*A*, the IL-12p40 panel and the IFN-beta panel were replaced and in Fig. 6*A*, the splices were indicated. This correction does not affect the results or conclusions of the study.

**Figure 5:**
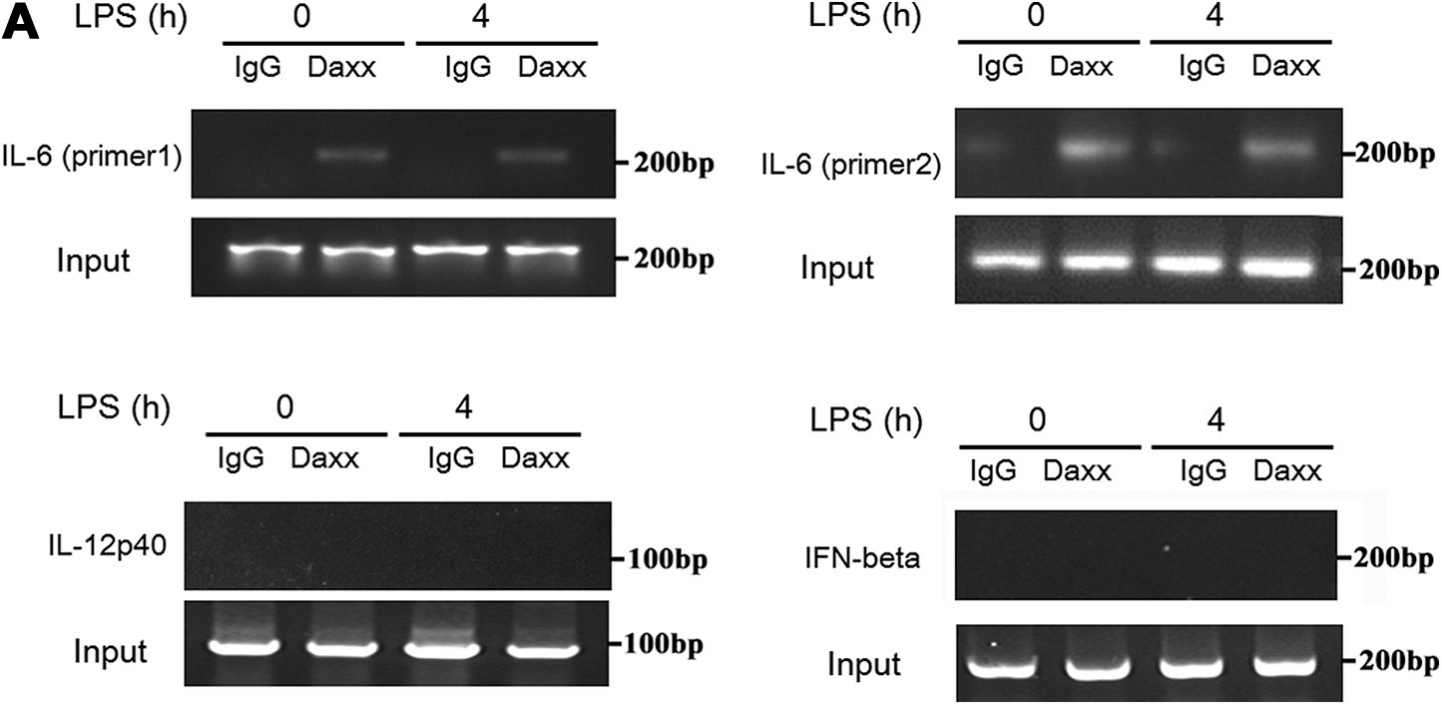

**Figure 6:**